# Leigh syndrome in individuals bearing m.9185T>C *MTATP6* variant. Is hyperventilation a factor which starts its development?

**DOI:** 10.1007/s11011-017-0122-1

**Published:** 2017-11-07

**Authors:** Dorota Piekutowska-Abramczuk, Rafał Rutyna, Elżbieta Czyżyk, Elżbieta Jurkiewicz, Katarzyna Iwanicka-Pronicka, Dariusz Rokicki, Sylwia Stachowicz, Joanna Strzemecka, Wiesław Guz, Michał Gawroński, Aneta Kosierb, Joanna Ligas, Mateusz Puchala, Anna Drelich-Zbroja, Małgorzata Bednarska-Makaruk, Wojciech Dąbrowski, Elżbieta Ciara, Janusz B. Książyk, Ewa Pronicka

**Affiliations:** 1 Department of Medical Genetics, The Children’s Memorial Health Institute, Aleja Dzieci Polskich 20, 04-730 Warsaw, Poland; 2 Chair and Department of Anaesthesiology and Intensive Therapy, Medical University of Lublin, Lublin, Poland; 3 Clinical Department of Child Neurology, Clinical Central Hospital No 2 in Rzeszow, Rzeszow, Poland; 4 Department of Radiology, The Children’s Memorial Health Institute, Warsaw, Poland; 5 Department of Audiology and Phoniatrics, The Children’s Memorial Health Institute, Warsaw, Poland; 6 Department of Pediatrics, Nutrition and Metabolic Diseases, The Children’s Memorial Health Institute, Warsaw, Poland; 7 Department of Neurology, Public Independent Clinic Hospital No 4 in Lublin, Lublin, Poland; 8 Department of Orthopaedics and Rehabilitation, Public Independent Clinic Hospital No 4 in Lublin, Lublin, Poland; 9 Institute of Health Sciences, Pope John Paul II State School of Higher Education, Biała Podlaska, Poland; 10 Department of Electroradiology, Institute of Nursing and Health Sciences, Faculty of Medicine, University of Rzeszow, Rzeszów, Poland; 11 Clinical Department of Radiology, Clinical Central Hospital No 2, Rzeszow, Poland; 12 Student Academic Club at The Chair and Department of Anaesthesiology and Intensive Therapy, II Faculty of Medicine with English Language Division, Medical University of Lublin, Lublin, Poland; 13 Department of Interventional Radiology and Neuroradiology Medical University of Lublin, Lublin, Poland; 14 Department of Genetics, Institute of Psychiatry and Neurology, Warsaw, Poland

**Keywords:** Leigh syndrome, m.9185T>C variant, *MTATP6*, Hypocapnia, Respiratory alkalosis, Oxygen therapy

## Abstract

Leigh syndrome (LS), subacute necrotizing encephalomyelopathy is caused by various genetic defects, including m.9185T>C *MTATP6* variant. Mechanism of LS development remains unknown. We report on the acid-base status of three patients with m.9185T>C related LS. At the onset, it showed respiratory alkalosis, reflecting excessive respiration effort (hyperventilation with low pCO_2_). In patient 1, the deterioration occurred in temporal relation to passive oxygen therapy. To the contrary, on the recovery, she demonstrated a relatively low respiratory drive, suggesting that a “hypoventilation” might be beneficial for m.9185T>C carriers. As long as circumstances of the development of LS have not been fully explained, we recommend to counteract hyperventilation and carefully dose oxygen in patients with m.9185T>C related LS.

**Electronic supplementary material:**

The online version of this article (10.1007/s11011-017-0122-1) contains supplementary material, which is available to authorized users.

## Introduction

Leigh syndrome (LS) is a progressive neurodegenerative disorder of genetic origin diagnosed on autopsy and/or with magnetic resonance imaging (MRI). It may be related to changes in over 75 various genes (Lake et al. 2016). One of recurrent causes of the late onset LS is m.9185T>C variant of the *MTATP6* gene (Moslemi et al. 2005). The reason for sudden development of LS in m.9185T>C carriers is not known, but an infection was underlined as a trigger in some reports (Pitceathly et al. 2012, Saneto and Singh 2010, Castagna et al. 2007). We assume that hypocapnia and respiratory (hypocapnic) alkalosis (Pronicka and Halikowski 1984; Pronicka 2017), reflecting hyperventilation, may contribute to LS occurrence. We established this on an empirical basis in three m.9185T>C carriers.

## Material and methods

### Patients with Leigh syndrome

Three patients with late onset LS were studied. There were two unrelated children and the mother of one of them bearing the *MTATP6* m.9185T>C variant, who developed LS at the age of 5, 9 and 33 years, respectively. Additionally, four m.9185T>C carriers without LS features, who were identified by cascade studies of both families were included in this study.

Details of the clinical course are presented in supplementary materials (Tables 1–3).

All procedures performed in the study were in accordance with the ethical standards of the CMHI bioethical committee and with the 1964 Helsinki declaration and its later amendments or comparable ethical standards.

Informed consent was obtained from all individual participants included in the study.

#### Family 1, patient 1

**Fig. 1:**
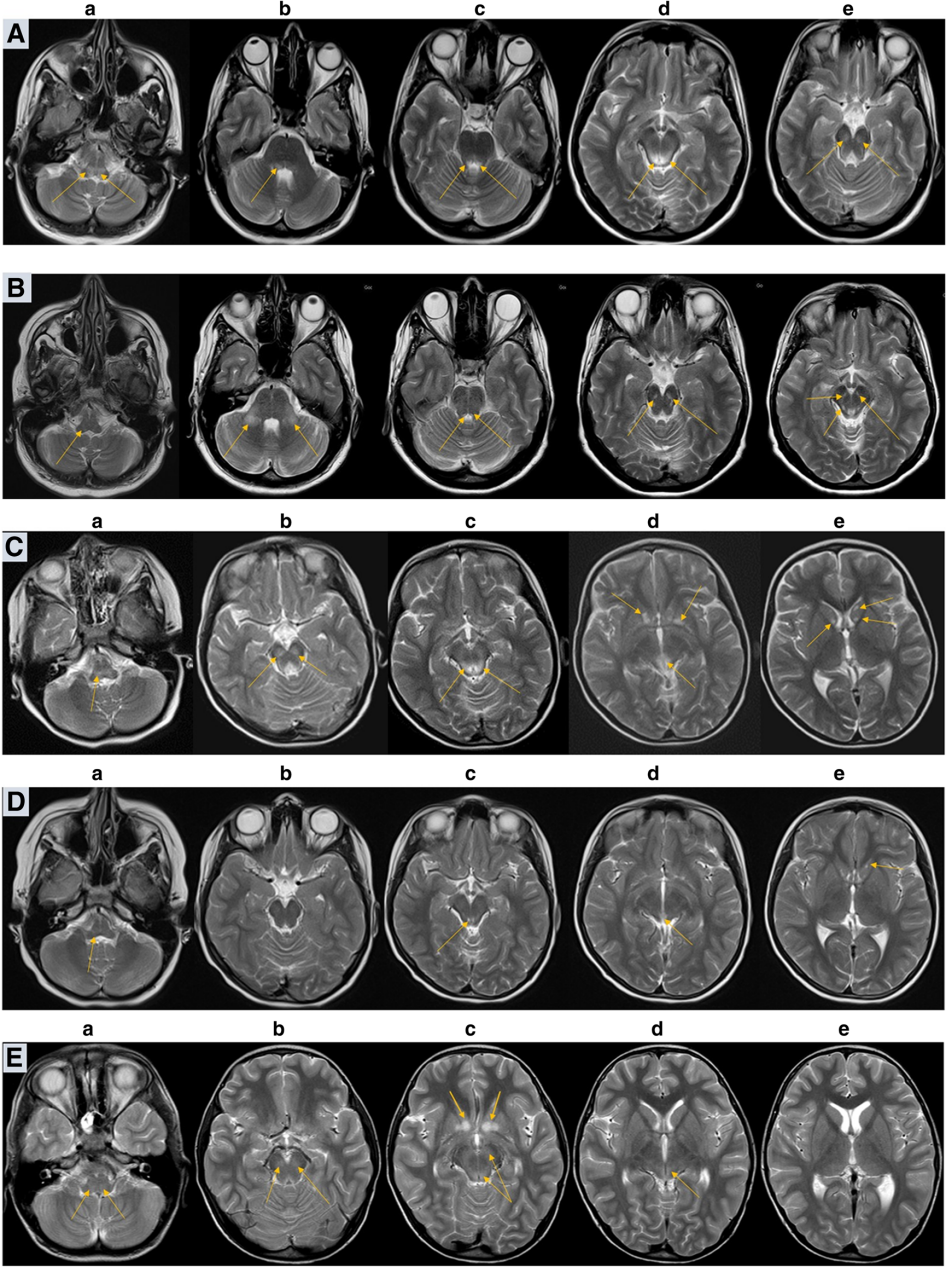

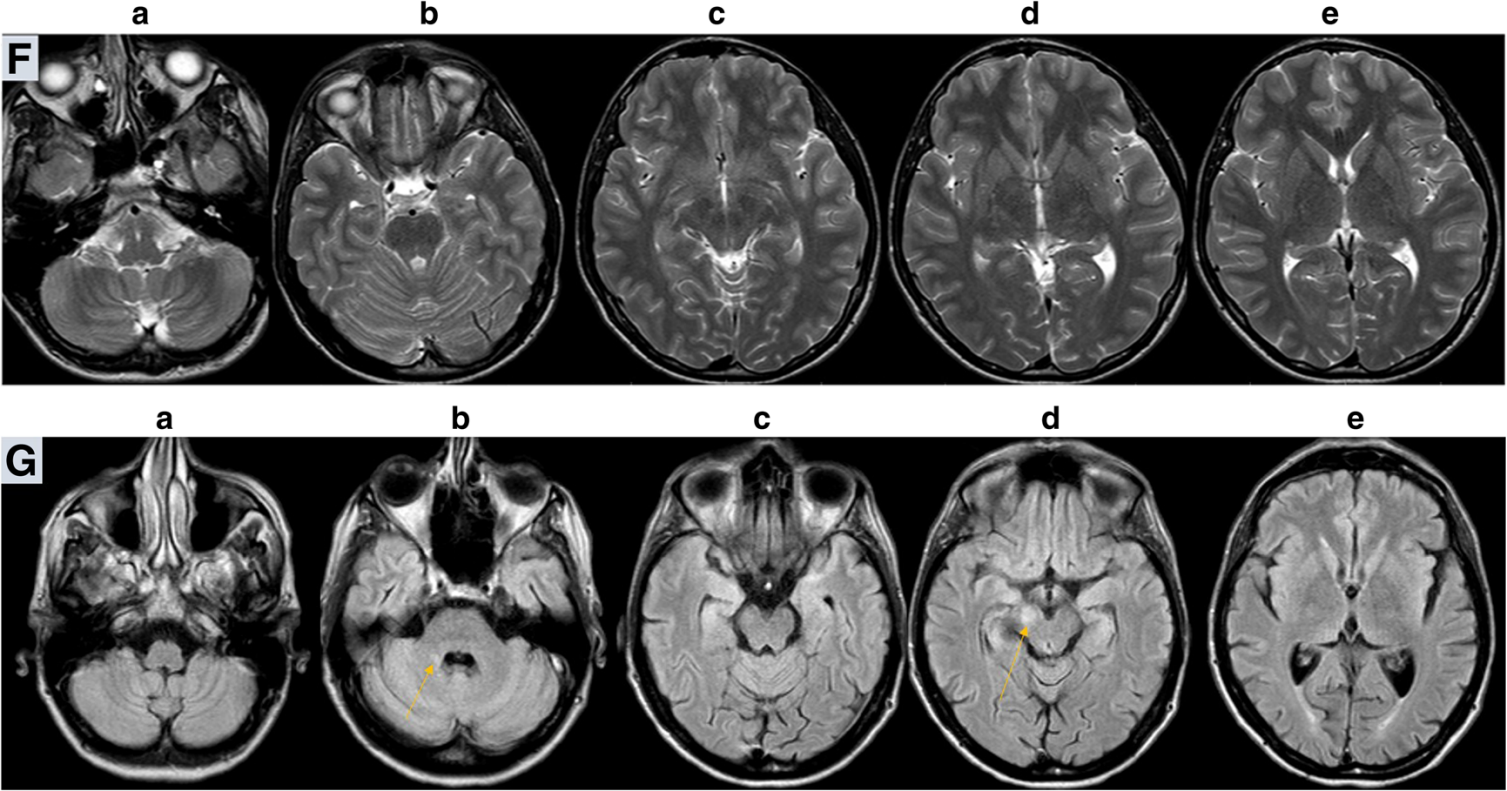
Serial axial T2-weighted brain MR images of patients 1, 2, 3 and family members (FLAIR sequence) bearing m.9185T>C variant of *MTATP6*, and autopsy findings in patient 3. Patient 1. Line A, at LS presentation at the age of 33: bilateral, symmetric hyperintensities in medulla oblongata, peripheral parts of pons and midbrain. Line B, on follow-up, 11 months later: a total regression of changes in peripheral parts of pons, presence of the previously detected lesions located in midbrain and in medulla oblongata. Greater degree of lesion demarcation. New symmetric lesions (T2-weighted hyperintensities) bilaterally within middle cerebellar peduncles. Patient 2. Line C, at LS presentation at the age of 5: high signal of the medulla oblongata (**a**), midbrain (**b**, **c**) and periaqueductal area (**d**). Small cystic-like lesions in the heads of the caudate nuclei (**e**). Line D, on the follow-up, at the age of 8: a partial but significant reduction in the severity of changes. Patient 3. Line E, at LS presentation at the age of 9: poor demarcation hyperintensities located in medulla oblongata (**a**), and midbrain (**b**, **c**, **d**); symmetric high signal lesions of the lower parts of striatum. Healthy brother of patient 3. Line F, normal MRI (**a**–**e**). Mother of patient 3. Line G, hyperintensive lesions in the posterior part of right middle cerebellar peduncle (**b**) and right cerebral peduncle (**d**), without changes in the medulla oblongata, periaqueductal region (**a**, **c**) and basal nuclei (**e**)

A 33 year-old woman, was admitted to the neurological ward because of exacerbation preceded by infection symptoms. Initially, a congenital polyneuropathy was diagnosed. In few days the patient developed severe respiratory problems. Late onset LS was diagnosed by MRI (Fig. 1, line A) and carriage of *MTATP6* m.9185T>C variant was established following the diagnosis in her daughter (patient 2). The patient needed artificial respiratory support and was hospitalized for 5 months.

On the follow-up examination, her clinical status was assessed as stabile. MRI performed after 11 months showed a partial regression of LS changes (Fig. 1, line B). Over a year since the LS onset, the patient was wheelchair depending, with good mental communication.

#### Family 1, patient 2

The daughter of patient 1, developed LS at the age of 5 (Fig. 1, line C). The only abnormality noted in the early childhood was a little sluggish speech. The course of LS was mild. Her clinical condition spontaneously returned to normal, she started primary school in the normal time (7 years). Control brain MRI performed at the age of 9 showed maintenance of previously seen LS changes, although in a less extent (Fig. 1, line D). At this time, molecular diagnostics revealed the *MTATP6* m.9185T>C pathogenic variant. Ascertain of the cause of LS in patient 2 coincided with the development of the similar disease in her mother (patient 1).

#### Family 2, patient 3

A 9 year-old boy was admitted to a regional hospital due to acute onset of imbalance, severe muscle weakness, fatigability, and sleepiness. MRI revealed LS changes (Fig. 1, line E) and the boy was referred to the referential centre for mitochondrial diagnostics.

**Fig. 2:**
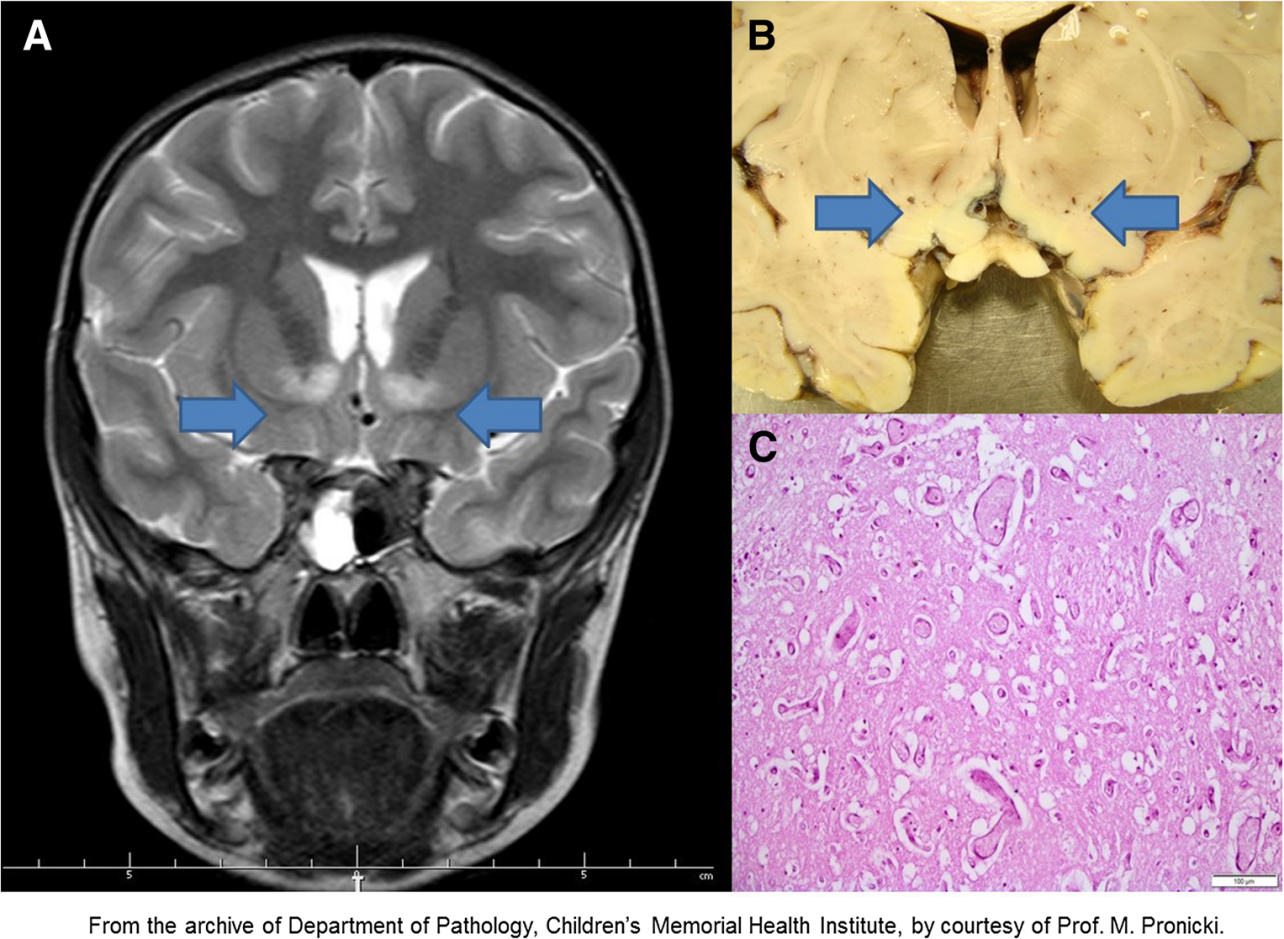
Brain autopsy of 9 year-old patient 3. Basal ganglia lesions, coronal section. Gross autopsy morphology (**b**) shows discrete symmetric discoloration of basal ganglia located in areas corresponding to MRI (**a**). Histopathological examination of affected areas reveal clearly pathological lesions, similar to that described in LS – mainly vascular capillary abundance, spongiosis, oedema (**c**: hematoxylin and eosin stain, original magnification 200X)

Shortly after admission, the boy collapsed suddenly and died in the episode of deterioration on the fourth day of hospitalization, within five weeks from the onset. Autopsy showed symmetrical changes characteristic for LS (proliferation of small vessels, spongiosis, damage to neurons) localized in periaqueductal areas and in the bottom of the fourth ventricle. The damage and loss of neurons were seen in dentate nucleus on both sides and to a less extent in olives nuclei. Location of brain changes on the autopsy entirely corresponded to previous MRI (Fig. 2). Accumulation of lipids in the muscle and liver as well as mosaic COX deficit in skeletal muscle were found. The *MTATP6* m.9185T>C variant was identified by subsequent next generation sequencing (NGS) (Pronicka et al. 2016).

### Characteristics of additional m.9185T>C carriers

Cascade molecular study of families 1 and 2, revealed four additional m.9185T>C carriers. The testing was positive in all relatives included in the search, with different heteroplasmy levels in their tissues (Supplementary Table 2).

In family 1, the m.9185T>C variant was found in the mother of patient 1, aged 61, (grandmother of patient 2), and in her second 32 year-old daughter (sister of patient 1). Their heteroplasmy levels in blood and urine were 45%, 55%, and 15%, respectively. No symptoms related to m.9185T>C carriage were revealed at careful examination (Supplementary Table 2).

In family 2, two carriers of m.9185T>C variant were identified (Supplementary Table 2). The older brother of patient 3, aged 18, was completely healthy despite >97% heteroplasmy level in blood. Brain MRI performed following the carriage detection was normal (Fig. 1, panel F). No abnormalities were revealed by extended audiological and otoneurological investigations (ABR, P300, VEMP, ENG).

The mother of two brothers from family 2 also showed m.9185T>C heteroplasmy >97% in blood and urine (Supplementary Table 2). She was healthy up to the age of 36. After death of her younger son, she developed dysarthria and depressive symptoms. At the age of 40, Alzheimer’s disease (AD) was diagnosed depending on positive four generation history of AD, and presence of *PSEN1* mutation previously identified in her son who died of LS (Pronicka et al. 2016). During observation, the patient developed clinical features atypical for AD (swallowing problems, seizures, spasticity, dystonia), but the brain MRI excluded LS (Fig. 1, panel G). No final conclusion could have been drawn on a possible modifying influence of m.9185T>C variant on the natural history of AD in this case.

### Measurement of blood gases

Acid-base status was repeatedly measured during hospitalizations in arterial, capillary or venous blood of the patients, the measurements were performed according to the instructions of the equipment manufacturers. GEM Premier 3500 gasometer made by Instrumentation Laboratory USA was used for patient 1 during her stay in the intensive care unit (ICU). In other cases, the measurements were performed in local laboratories and the type of equipment used was not possible to establish.

The values of pH, pCO_2_ and pO_2_ were measured directly in the apparatus. Concentration of HCO^−^_3_ was calculated from the received data, in relation to the atmospheric pressure and temperature. HCO^−^_3_ values were not checked with biochemical methods. Control values available in the literature references were applied for data interpretation (Gomella and Haist 2002).

### Heteroplasmy analysis

Heteroplasmy levels for the *MTATP6* m.9185 T > C variant stated in this study and reported in the literature references were analysed as a whole with respect to the age of onset of the disease, symptoms and outcome.

## Results

### Acid-base balance findings

**Fig. 3:**
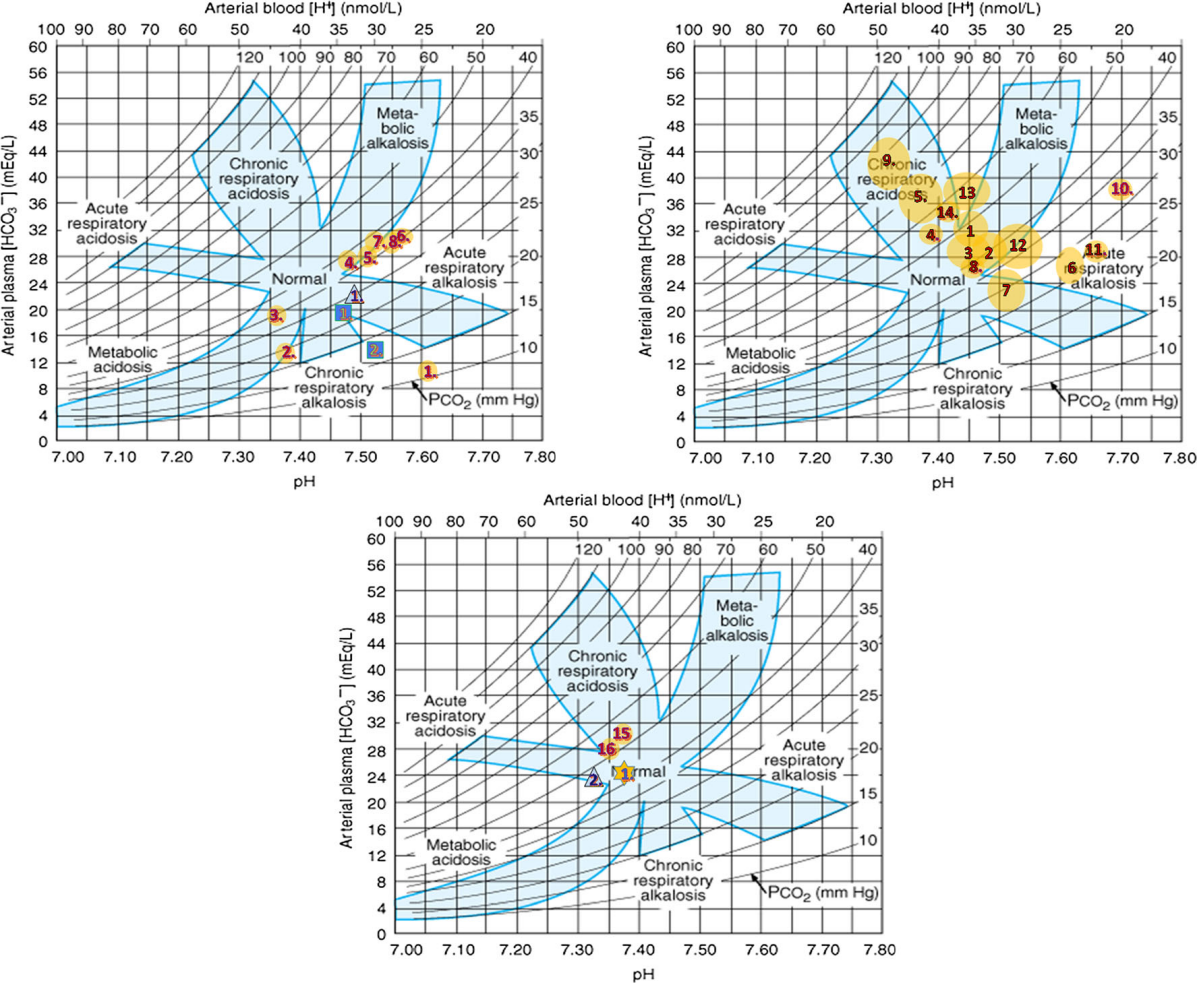
Changes of acid base data observed in reported carriers of m.9185T>C variant. **a** During spontaneous breathing in patient 1 (circles), patient 2 (triangle) and patient 3 (squares). **b** On artificial ventilation in patient 1 (circles). **c** During recovery of patient 1 (circles) and patient 2 (triangle), and in asymptomatic brother of patient 3 (star). Explanation of numbers inside symbols: For patient 1, one number corresponds to many tests performed during the entire observation, whose results were situated in the same position of the nomogram; 53 measurements were included together and clustered/gathered in circles 1–16 (Supplementary Table 1, column 1). For other three individuals (triangles, squares, star), the numbers show order in time of measurements. Copyright/licensed by McGraw-Hill Education adopted from Gomella and Haist (2002)

In patient 1, at the beginning, acid-base data showed a substantial respiratory alkalosis, with pH 7.59 (ref. < 7.43), pCO_2_ 11.2 mmHg (ref. > 35), and HCO^−^_3_ 10.4 mmol/l (ref. > 20), which reflected her persisting hyperpnea (Table 1, Fig. 3, panel A, point 1). At that time, high pO_2_ pressure of 148.4 mmHg (ref. < 100) was found. The patient was breathing on her own and received passive oxygen therapy (FiO_2_ = 0.35).

**Table 1:** Acid-base parameters of three spontaneously breathing individuals at presentation of Leigh syndrome (patients 1, 2 and 3)

Parameter	Patient 1*	Patient 2	Patient 3	Control range (Gomella and Haist 2002)
Age	5 years	36 years	9 years	
Time	first day	first days	first two days	
Sampling	capillary blood	ND	ND	
pH	7.59	7.49, 7.46, 7.463	7.47, 7.52, 7.53	7.37–7.44 (a) 7.31–7.41(v)
pCO_2_ mmHg	11.2	27.8, 30.2, 29	27, 17.1, 18	36–44 (a) 40–52 (v)
HCO^−^_3_ (ecf) mmol/L	10.4	23.9, 23.4, 23.0	23.6, 13.5, 14.6	22–26 (a) 22–28 (v)
BE mmol/L	11.3	−1.9, −2.1, −2.8	−2.7, −6.2, −5.1	0 ± 2 (a, v)
pO_2_ mmHg	148.4	53.6	76, 51.9, 78.7	80–100 (a) 30–50 (v)
O_2_ saturation %	99	92.9, 95.8, 91	98	>95 (a) 60–85 (v)
Interpretation	respiratory alkalosis, partially compensated	respiratory alkalosis	respiratory alkalosis, start of metabolic compensation	–

*on passive oxygen therapy (nasal cannula)

Legend: pCO_2_: partial pressure of carbon dioxide; pO_2_: partial pressure of oxygen; HCO^−^_3_: bicarbonate concentration; BE: base difference (deficit/excess); ecf: extracellular fluid; (a): arterial, capillary blood; (v): venous blood; ND: not done

HCO^−^_3_ and BE values are calculated using the Henderson-Hasselbach equation

When the patient was placed on artificial ventilation support at intensive care unit, an uncommon pattern of acid-base equilibrium was observed, with mean pH 7.47 ± 0.08 (ref. 7.37–7.44), pCO_2_ 43.6 ± 13.8 mmHg (ref. 24–44), HCO^−^_3_ 30.4 mmol/l (ref. 22–26) in arterial blood, with a shift to the “metabolic alkalosis” field on the nomogram of acid base disorders (Fig. 3, panel B, points 1–4, 8, 12). This tendency was observed as the main feature during the entire stay at the ICU. Unfortunately, mechanisms of renal compensation were not studied in detail.

A few times, during attempts of reduction of mechanical ventilation, the retention of CO_2_ occurred, resulting in the transient “respiratory acidosis” status (Fig. 3, panel B, points 5, 9). Episodes of excessive ventilation resulting in respiratory alkalosis pattern were noticed a few times (Fig. 3, panel B, points 6, 7, 10, 11). Only when “adequate” mechanical ventilation was restored, the arterial blood gases balance returned to the lower pCO_2_ range and the “metabolic alkalosis” field of the chart. During observation, at various points of artificial ventilation maintenance, a disproportionally low pCO_2_ (of about 1–2 mmHg) for pH/ HCO^−^_3_ (calculated values for HCO^−^_3_) could have been read out on the nomogram.

After efficient withdrawal of artificial ventilation, the pattern of “metabolic alkalosis” maintained for a few days (Fig. 3, panel B, points 13, 14). The respective values were: 7.45 ± 0.02 (ref. 7.37–7.44), pCO_2_ 52.1 ± 2.1 mmHg (ref. 24–44) and HCO^−^_3_ 36.7 ± 2.2 mmol/l (ref. 22–26). Without oxygen therapy, the arterial blood values of pO_2_ decreased to mean 84.3 ± 11.0 mmHg (*n* = 7, ref. 80–100).

After discharge, and during several months of clinical recovery, the status of the acid-base balance was consequently maintained at the borderline of “chronic respiratory acidosis”. Respective results for venous blood were as follows: pH 7.37, 7.35 (ref. 7.31–7.41), pCO_2_ 53.9, 51.6 mmHg (ref. 40–52), HCO^−^_3_ 26.7, 28.6 mmol/l (ref. 22–28), which represented a suppression of respiratory effort (a peripheral neuropathy contribution cannot be excluded). We may assume that such pattern of acid-base balance (Fig. 3, panel C) may be optimal for long term wellbeing of patient 1.

In patient 2, the acid base balance was assessed three times at LS presentation. The data indicated respiratory alkalosis (Table 1, Fig. 3, panel A, triangles). Recovery was achieved spontaneously; only one gasometric data is available for that period (Fig. 3, panel C, triangle).

In patient 3, hyperventilation without any signs and symptoms of upper respiratory tract infections was mentioned in the first note taken on the physical examination. Respiratory alkalosis was demonstrated three times during two first days of hospitalization (Table 1, Fig. 3, panel A, squares). It switched to metabolic acidosis just before the boy’s sudden death (venous blood: pH 7.228, pCO_2_ 29.1 mmHg, HCO^−^_3_ 13.5 mmol/l, BE −14.4 mmol/l, pO_2_ 72.1 mg%, O_2_ saturation 82,2%, ion gap 37.5 mmol/l, ref. see Table 1).

In the healthy brother of patient 3, asymptomatic m.9185T>C carrier, the acid base balance subsequently tested three times was at the normal range (mean pH 7.385, pCO_2_ 44.6 mmHg, HCO^−^_3_ 23.2 mmol/l, ion gap 5.4 mmol/l) (Fig. 3, panel C, star), as well as lactate concentration (mean 16.1 mg/dl, *n* = 3, ref. < 20 dl). No blood gases data was available for the AD mother of patient 3.

### Heteroplasmy of m.9185T>C variant versus symptomatology

The heteroplasmy levels found in this study and reported in the literature references are summarized in Table 2, in relation to the disease onset, studied tissue, LS occurrence and outcome. The analysed material in total includes the data of 81 individuals from 16 families aged 0.5 to 70. Heteroplasmy level was assessed for the first time during childhood in 13 patients, during adolescence in 5 patients and at adult age in the remaining individuals (Supplementary Table 2).

**Table 2:** Clinical characteristics of heteroplasmy levels in 81 individuals with *MTATP6* m.9185T>C variant

	Mean +/− SD	Median value	Range	Number of data*
Onset of symptoms, years	23.6 ± 19.3	18	0.5–80	56
Leigh syndrome, years	12.1 ± 9.5	9	0.5–34	18
Last seen/death, years	29.3 ± 18.2	24	7–79	33
Age of death, years	15.9 ± 13.6	10	0.5–48	11
Heteroplasmy study, years	18.1 ± 14.6	16	7–61	27
Blood, %	85.5 ± 21.7	97	15–100	44
Urine, %	83.4 ± 21.8	91	15–100	17
Buccal cells, %	93.6 ± 9.2	98.5	67–100	14
Hair, %	85.9 ± 17.8	91.5	35–100	14
Fibroblasts, %	94.1 ± 4.9	91	90–100	7
Muscle, %	95.9 ± 7	99	73–100	18
All tissue minimal value, %	86.9 ± 21	97	15–100	75
All tissue maximal value, %	90.3 ± 19.6	100	15–100	75

Literature data. (Moslemi et al. 2005, Castagna et al. 2007, Childs et al. 2007, Saneto and Singh 2010, Pitceathly et al. 2012, Brum et al. 2014, Danqun et al. 2015 abstr., Pronicka et al. 2016). More details: Supplementary Table 2

*in 28 patients heteroplasmy was measured in DNA specimens isolated from more than one tissue

Most of the patients presented the first symptoms, usually of mild severity, during childhood. The disease was slowly progressive and led to wheelchair dependence in late adulthood. Life expectancy does not seem to have been shortened. Various diagnoses including: CMT t.2, intermittent ataxia, MND, SCA, NARP, peripheral neuropathy, polyneuropathy, periodic paralysis, gait imbalance, pes cavus, were established in the reported patients (Supplementary Table 2).

LS developed in 21 m.9185T>C carriers (25.9%). LS symptoms usually superimposed over earlier present neurological disturbances. LS beginning phase was acute or subacute, and disease developed in the age of 5–34, frequently in the course of infection. Asymptomatic carriage was rare (17.3%) and mainly concerned the cases with low heteroplasmy level (Supplementary Table 2).

## Comments and discussion

In three patients with LS of late onset (5–33 years), bearing *MTATP6* m.9185T>C variant, we showed (on empirical basis) that hyperventilation coincided with LS onset in all of them. No such coexistence was reported previously, with one exception (Castagna et al. 2007). However, many other factors, coexisting with clinical deterioration of m.9185T>C carriers were reported. The set includes febrile viral illness (Castagna et al. 2007, Saneto and Singh 2010, Pitceathly et al. 2012), summer heat (Saneto and Singh 2010), panic attacks (Childs et al. 2007), prolonged sitting (Auré et al. 2013) (Supplementary Table 3).

Excessive respiration effort (hyperventilation) in any case causes changes in partial blood gases pressure (p). If hyperventilation persists, it leads to a disproportionally low pCO_2_ (hypocapnia). Blood pO_2_ independently adjusts to the composition of inspiratory air. We noticed such changes in blood gases in reported patients at onset of LS, with low pCO_2_ in patients 1, 2 and 3, and relatively high pO_2_, especially in patient 1, during passive oxygen therapy (Table 1). Occurrence of hyperventilation was reflected by acid-base pattern, which shifted to the “respiratory alkalosis” field on the nomogram for acid–base disorders (Fig. 3, panel A). The alkalosis was initially acute (non-compensated, with blood pH maximum of 7.59 (ref. < 7.43) and later compensated, due to the start of renal HCO^−^_3_ loss. Lowering of HCO^−^_3_ was always preceded by lowering of pCO_2_.

Our study confirms that the course of m.9185T>C related LS differs markedly from the majority of other LS types, in which the onset is early, and outcome irreversibly poor. The m.9185T>C-related LS tends to recover, at least partially. The natural history of the condition in some respect resembles a common m.8993 T > C substitution in *MTATP6* (Craig et al. 2007). Two out of three reported LS patients with m.9185T>C variant recovered partially (patient 1) or almost completely (patient 2). The recovery of m.9185T>C-related LS was previously reported at least in nine patients (Moslemi et al. 2005; Childs et al. 2007; Saneto and Singh 2010) (Supplementary Table 3).

Analysis of changes in acid-base parameters during the course of LS in surviving patients 1 and 2 shows that their recovery coexisted with the higher pCO_2_ (“hypercapnia”) and lower pO_2_ (“hypoxia”) values (Fig. 3, panel C), in contrast to the deterioration period (Fig. 3, panel A). Surprisingly, this data points to a relatively low respiratory drive on the recovery. It may be speculated that the m.9185T>C carriers do not require increased oxygen concentrations, if compared with healthy controls, and that higher CO_2_ and lower O_2_ supplies may be beneficial for them.

The above data is in accordance with our “hypocapnic hypothesis” of LS (Pronicka 2017). The hypothesis assumes that hypocapnia (low pCO_2_) leads to intracellular alkalization (high pH) of brain cells. Inside mitochondria, the signal of high pH/low bicarbonate ion (HCO^−^_3_) is transmitted by soluble adenyl cyclase (sAC) through cAMP dependent manner (Buck and Levin 2014), and results in modification of activities of proteins involved in OXPHOS, apoptosis and other relevant processes. It can initiate brain lesions (necrosis, apoptosis, hypervascularity) in *MTATP6* mutated cells (astrocytes?) residing at the LS area of the brain.

The hypocapnic hypothesis of LS, and our findings that the patients with m.9185C > T-related LS showed clinical improvement in parallel with higher pCO_2_ and lower pO_2_, may also help to understand the basis of beneficial effect of hypoxia in animal model of LS (Jain et al. 2016). It has been shown that chronic hypoxia (11% vs 34%) in surrounding atmosphere leads to a marked improvement in survival, body weight, body temperature, behavior, neuropathology and disease biomarkers in the *NDUFV4* mutated mouse model (Jain et al. 2016).

The analysis of the patients included in the study and those previously reported in the literature (in total number of 81), extends our knowledge on natural history of carriage of m.9185T>C variant in *MTATP6*. It shows that the m.9185T>C carriage alone does not prejudge the occurrence of disease symptoms, including LS. About 18% of carriers may remain healthy throughout life, particularly if the m.9185T>C heteroplasmy is low. However, in most of the individuals, discrete anomalies of development appear during early childhood (Supplementary Table 3). Later in life, the carriers developed neuropathic symptoms recognized as CMT1 (Pitceathly et al. 2012), periodic paralysis (Auré et al. 2013), peripheral neuropathy and others.

LS occurred in about one quarter of carriers, including our patients 1, 2 and 3, always of the late-onset type and at the age difficult to predict. It seems that there are three intervals of increased risk of LS: 5–9 years of age, 16–19, and above 30. The onset was acute or subacute (Moslemi et al. 2005, Pitceathly et al. 2012, Saneto and Singh 2010, Castagna et al. 2007, Auré et al. 2013, Brum et al. 2014, Pfeffer et al. 2012, Childs et al. 2007, Danqun et al. 2015, this study). At present, it is difficult to conclude whether the m.9185T>C variant on its own may be the “isolated” cause of LS.

It is known of course that high level of heteroplasmy constitutes an important risk factor for appearance of symptoms (Table 2). However, even 100% of mutated mtDNA does not prejudge the disease onset like in the 18 year-old asymptomatic brother of patient 3. We suppose that extra harmful factors have to occur (as discussed above hyperventilation evoked by a stressful event) to induce a chronic neuropathy or LS in m.9185T>C carriers.

Summing up, at present there is no basis to unambiguously answer the question whether being an m.9185T>C carrier is equal with having a mitochondrial disease (with a delayed onset). To extend our knowledge of the natural history of the m.9185T>C carriage, the population prevalence and prospective observations of asymptomatic carriers throughout their lives are desired.

Better understanding of the natural history of m.9185T>C carriage, as well as pathomechanism of LS development, will allow to find out if there is any way to prevent m.9185T>C related symptoms in asymptomatic carriers, as well as occurrence of LS in patients with neuropathy associated with this *MTATP6* variant.

As long as it is not fully explained, basing on our own observations, we suggest to counteract hyperventilation (hypocapnia) and carefully dose oxygen in m.9185T>C carriers and the patients with m.9185T>C related LS.

## Electronic supplementary material

ESM 1 (DOCX 58 kb)

ESM 2 (DOCX 115 kb)

ESM 3 (DOCX 113 kb)
