# Supplementary material for: Leigh syndrome in individuals bearing m.9185T>C MTATP6 variant. Is hyperventilation a factor which starts its development?
Source: Metab Brain Dis. 2017 Nov 7;33(1):191–9. doi: 10.1007/s11011-017-0122-1 (PMC5769826; doi:10.1007/s11011-017-0122-1)
Supplement: Supplementary file 1 — (DOCX 58 kb) [file 11011_2017_122_MOESM1_ESM.docx]

Supplementary Table 1. Selected data from medical records of patient 1 during her stay on neurology and ICU (details for Table 2 and A, B panels of Figure 2 in the main text).

| **I hospitalisation in Clinic of Neurology, Independent Public Clinical Hospital N^o^ 4 in Lublin,** | | |
| --- | --- | --- |
| **Date** | **Clinical status** | **Other** |
| **16.11.2015–23.11.2015** | Female patient, age 33, admitted to the hospital due to significant intensification of continuing since childhood difficulties in walking and the sensation of ”heavy legs” (for the last 5 weeks), exacerbation of symptoms after sinusitis (treated with amoxicillin with clavulanic acid).  Neurological examination: Patient in a good general condition, conscious, logical reasoning retained. Narrow palpebral fissures, slight nystagmus when looking left. Paraparesis, decreased deep reflexes, plantar reflexes absent. Independent gait, with lameness, hammer toes (since childhood).  Romberg’s test: unstable balance.  Initial diagnosis: Congenital peripheral neuropathy of unknown cause | EMG: changes characteristic for sensorimotor demyelinating polyneuropathy decreased or increased in the area of lower limbs, with no conduction blocks present. |

| **II hospitalization in Clinic of Neurology, Independent Public Clinical Hospital N^o^ 4 in Lublin, 06.12.2015–16.12.2015** | | | | |
| --- | --- | --- | --- | --- |
| Type of acid-base profile; interpretation  *(see point number on Figure 1A/B)* | Day of hospitalization; time of blood sampling | Acid-base data | Clinical status | Other results, treatment |
|  | 1 day  (06.12.2015) |  | Female patient admitted to the hospital due to an attack of dyspnoea, weakness, ptosis, continued since the day before admission. Directed to the Clinic of Neurology in Lublin with the diagnosis of the myasthenic syndrome. |  |
| 1.  acute > chronic respiratory alkalosis) | 2. day  (7.12.2015) | pH = 7.59 pCO_2_ = 11.2 mmHg HCO_3_ = 10.4 mmol/L pO_2_ = 148.4 mmHg  BE (ecf) = 11.3 mmol/L | Temperature up to 37.4°C, this day only.  Spontaneously breathing patient with oxygen therapy (FiO_2_ = 0.35). | troponin I: 0.024 ng/ml  EMG (7.12.2015): incorrect result of the electrical stimulation muscle weakness test, disorders of the postsynaptic nature. Slight degree of deviations did not, however, explain existing breathing disturbances and the general clinical condition of the patient. |
|  | 3 day (08.12.2015) |  | Supratentorial, weakly isolated, symmetric areas of elevated signals on T2-weighted images located entrally and in the posterior of the midbrain, in the peripheral part of the pons and centrally and slightly more peripherally in medulla oblongata.  After administration of the gadolinium-based contrast, discreet post-contrast enhancement in areas of increased signal intensity in medulla oblongata. | leukocytosis: 19.59 K/uL  Brain MR (8.12.2015): inconclusive image, changes in pons and medulla oblongata might suggest: Leigh syndrome, ADEM (Acute Demyelinating Encephalomyelitis), inflammatory. |
|  | 4 day (09.12.2015) |  |  | lactic acid = 2.0 mmol/L |
|  | 5 day 10.12.2015 |  | Shallow breath.  Until now mobile (walked poorly), have used the wheelchair.  Talked, reported dyspnoea. | leukocytosis: 21.53 K/uL |
| 2.  3.  (compensation: increased HCO_3_, decreased hyperventilation, no metabolic acidosis) | 6 day 11.12.2015 | 2. Time: 17:13 pH = 7.35 pCO_2_ = 25.2 mmHg HCO_3_ = 13.6 mmol/L  pO_2_ = 83 mmHg  BE (ecf) = 12 mmol/L  3. Time: 23:12 pH = 7.33 pCO_2_ = 38.3 mmHg HCO_3_ = 19.7 mmol/L  pO_2_ = 148.4 mmHg  BE(ecf) = 11.3 mmol/L | Significant deterioration in the patient’s condition. Increased dyspnoea. Spontaneously breathing patient with oxygen therapy (FiO_2_ 0.35 to 0.45). | troponin I: 0.462 ng/ml  Time 17:04  troponin I: 2.53 ng/ml |
| 4.  5.  (compensation continued, metabolic adaptation) | 7 day 12.12.2015 | 4. Time: 09:25 pH = 7.45 pCO_2_ = 39.6 mmHg HCO_3_ = 27 mmol/L  pO_2_ = 61.1 mmHg  BE(ecf) = 3 mmol/L  4. Time: 19:44 pH = 7.49 pCO_2_ = 37 mmHg HCO_3_ = 27.8 mmol/L  pO_2_ = 210.4 mmHg  BE(ecf) = 4.5 mmol/L | From 12 noon to 4 p.m. temperature up to 38.9°C.  Unconscious, tachycardia, SpO_2_ 87–94%.  CT: pulmonary embolism excluded.  Increased left atrium and left heart ventricle.  Pulmonary oedema/alveolar haemorrhage.  Massive left lung parenchymal densities of inflammatory nature, ground glass opacities, supradiaphragmatic and in the right lung apex.  Time 14:10: **INTUBATED**, mechanical ventilation applied.  **DIAGNOSIS:**  **PNEUMONIA CIRCULATORY FAILURE PULMONARY OEDEMA** | troponin I: 5.168 ng/ml troponin I: 3.6 ng/ml  NT-proBNP: 11550 pg/ml CRP: 43.385 mg/l WBC: 40 K/uL procalcitonin: 0.38 ng/ml |
|  | 8 day 13.12.2015 |  | **Intubated, mechanical ventilation applied.** | WBC: 22.04 K/uL D-dimer: 1662 ng/ml CRP: 23.905 mg/l |
| 6. | 9 day 14.12.2015 | pH = 7.54 pCO_2_ = 37.5 mmHg HCO_3_ = 31.3 mmol/L  pO_2_ = 228.1 mmHg  BE(ecf)= 8.7 mmol/L  Metabolic alkalosis | **Intubated, mechanical ventilation applied.** | WBC: 12.06 K/uL D-dimer: 1398 ng/ml CK: 278 U/L >> 209 U/L CK-MB: 20.1 U/L >> 15.9 U/L  troponin I: 0.487 ng/ml  lactic acid: 2.2 mmol/L (19.8 mg/dl) |
| 7. | 10 day 15.12.2015  (metabolic alkalosis) | pH = 7.50 pCO_2_ = 39 mmHg HCO_3_ = 29.9 mmol/L  pO_2_ = 158.5 mmHg  BE(ecf) = 6.8 mmol/L | **Intubated, mechanical ventilation applied.** | **Lab tests**:  CK: 141 U/L troponin I: 0.206 ng/ml NT- proBNP: 334.6 pg/ml  **EEG**:  In the frontal-parietal-temporal lobe area, individual, series or groups of slow waves (delta–theta: 1.5–2,4 Hz, amplitude: 60–90–120 uV) standing out against the systolic activity.  **Echocardiography**:  within normal range. |
| 8. | 11 day 16.12.2015  (metabolic alkalosis) | pH = 7.52 pCO_2_ = 37.6 mmHg HCO_3_ = 30.6 mmol/L  pO_2_ = 103.7 mmHg  BE(ecf) = 7.9 mmol/L | **Intubated, mechanical ventilation applied.**  **Squeezes hand on request.** | WBC: 17.1 K/uL  lactic acid: 1.5 mmol/L (13.5 mg/dl) |

| **ICU, Independent Public Clinical Hospital N^o^ 4 in Lublin, 16.12.2015–16.02.2016** | | | | |
| --- | --- | --- | --- | --- |
| **N^o^** | **Date** | **Gasometry** | **Clinical status** | **Other** |
| 1. | 11 day, 16.12.2015  (metabolic alkalosis)  (slightly too low CO_2_ = 2–3 mmHg) | pH = 7.44 pCO_2_ = 46 mmHg HCO_3_ = 31.2 mmol/L  pO_2_ = 195 mmHg  BE(ecf) = 7.0 mmol/L | Intubated, mechanical ventilation applied.  On admittance, the patient conscious, basic verbal contact maintained. Respiratory failure, intubated, mechanical ventilation applied, FiO_2_ = 0.35, PEEP = 5. **No own respiratory drive???**  **oxygen = 35%**  Repeatable low grade fever, own diuresis efficient. Enteral nutrition.  Patient hemodynamically stable.  Pupils equal and reactive to light. Ptosis.  Quadriparesis especially pronounced in the area of lower limbs.  SAPS II: 34; 15,3%  APACHE II: 18; 29,1%  SOFA: 4; <10% |  |
| 1. | 12 day, 17.12.2015  (metabolic alkalosis)  (slightly too low CO_2_ = 2–3 mmHg) | pH = 7.46 pCO_2_ = 46 mmHg HCO_3_ = 32.7 mmol/L  pO_2_ = 117 mmHg  BE(ecf) = 8.9 mmol/L | **Intubated, mechanical ventilation applied.** |  |
| 2. | 13 day, 18.12.2015, 19:46  (metabolic alkalosis) | pH = 7.47 pCO_2_ = 40 mmHg HCO_3_ = 29.1 mmol/L  pO_2_ = 177 mmHg  BE(ecf) = 5.4 mmol/L | **Intubated, mechanical ventilation applied.**  Tracheotomy. Following the procedure patient ventilated in SIMV 35% oxygen. |  |
| 3. | 14 day, 19.12.2015, 15:19  (metabolic alkalosis) | pH = 7.45 pCO_2_ = 41 mmHg HCO_3_ = 28.5 mmol/L  pO_2_ = 163 mmHg  BE(ecf) = 4.5 mmol/L | Patient critical but stable. Sedated but easy to wake. Contact possible to establish. On request squeezes both hands, left hand weaker. Moves toes of both lower limbs. Significant weakening of muscle strength visible. Tracheostomy. Artificial lung ventilation in SIMV mode, **no own breathing**. FiO_2_ = 0.5. Cardiovascular system stable. | Dexdor (dexmedetomidine) 2% Propofol (Diprivan) no amines no antibiotics |
| 4. | 15 day, 20.12.2015, 21:55  (respiratory alkalosis)  reducing ventilator support | pH = 7.39 pCO_2_ = 52 mmHg HCO_3_ = 31.5 mmol/L  pO_2_ = 161 mmHg  BE(ecf)= 6.5 mmol/L  **lactates = 0.5 mmol/L** | Patient sedated but contact possible to establish. Cardiovascularly stable. **Ventilation mode changed into proportional assist ventilation (PAV), with good tolerance**. | *Decrease respirator, weak muscles???* |
| 5. | 16 day, 21.12.2015, 07:28  ( respiratory acidosis - reducing ventilator support) | pH = 7.37 pCO_2_ = 61 mmHg  HCO_3_ = 35.3 mmol/L  pO_2_ = 159 mmHg  BE(ecf)= 10.0 mmol/L  **lactates = 0.3 mmol/L** | Following reduction in sedation the patient conscious, but unwilling to establish contact. Auscultatory examination detects correct vesicular murmur. Patient ventilated in CPAP mode with PS and 35% oxygen in respiratory mixture. Attempt at PAV unsuccessful, significant **tachypnea and retention of**  CO_2_. Enteral nutrition, industrial diet. Temperature up to 38°C. |  |
| 6 | 16 day, 21.12.2015, 15:16  (acute respiratory alkalosis)  increasing ventilatory support | pH = 7.62 pCO_2_ = 26 mmHg  HCO_3_ = 26.7 mmol/L  pO_2_ = 129 mmHg  BE(ecf) = 5.5 mmol/L |  |  |
| 7 | 16 day, 21.12.2015, 22:42  (acute respiratory alkalosis)  – increased ventilatory support | pH = 7.51 pCO_2_ = 30 mmHg  HCO_3_ = 23.9 mmol/L  pO_2_ = 109 mmHg  BE(ecf) = 0.9 mmol |  |  |
| 7. | 17 day, 22.12.2015, 14:20  (acute respiratory alkalosis) | pH = 7.52 pCO_2_ = 28 mmHg  HCO_3_ = 22.9 mmol/L  pO_2_ = 151 mmHg  BE(ecf) = 0.0 mmol/L | Patient sedated, but contact possible to establish. Cardiovascularly stable. Patient ventilated in CPAP mode with PS and 35% oxygen in respiratory mixture, temperature: 38.2°C. | creatinine = 0.4 mg/dL urea = 32.4 mg/dl |
| 7. | 18 day, 23.12.2015, 15:18  (acute respiratory alkalosis) | pH = 7.51 pCO_2_ = 27 mmHg  HCO_3_ = 21.5 mmol/L  pO_2_ = 171 mmHg  BE(ecf) = -1.5 mmol/L | Patient sedated, but contact possible to establish. Tracheostomy. Patient ventilated in CPAP mode with PS and 35% oxygen in respiratory mixture. Cardiovascularly stable. Abdomen soft, painless. |  |
| 7. | 19 day, 24.12.2015, 22:20  (acute respiratory alkalosis)  reducing ventilator support | pH = 7.49 pCO_2_ = 29 mmHg  HCO_3_ = 22.1 mmol/L  pO_2_ = 176 mmHg  BE(ecf) =  -1.2 mmol/L | The patient’s condition seems to improve. Muscle strength improves. Today the patient respired in the **automatic tube compensation (ATC) mode, with no retention of carbon dioxide**. Conscious, limited sedation, enteral nutrition, industrial diet. Fever absent. |  |
| 7. | 20 day, 25.12.2015, 14:52  Acute respiratory alkalosis - spontaneously breathing patient | pH = 7.51 pCO_2_ = 30 mmHg  HCO_3_ = 23.9 mmol/L  pO_2_ = 145 mmHg  BE(ecf) = 0.9 mmol/L | Fever absent. **In the morning patient weaned from respirator and maintained on passive oxygen therapy with a nasal cannula**. |  |
| 8. | 21 day, 26.12.2015  Normalization of blood gases - spontaneously breathing patient | pH = 7.46 pCO_2_ = 36 mmHg  HCO_3_ = 25.6 mmol/L  pO_2_ = 100 mmHg  BE(ecf) = 1.8 mmol/L | Conscious, limited sedation. Before noon infusion of sedatives terminated. **The patient breathes on her own via the tracheostomy with passive oxygen therapy. Moves her hands and legs, however muscle strength weakening still noticeable**. Abdomen soft, painless, peristalsis present. Efficient diuresis. Enteral nutrition, industrial diet. Temperature up to 38.1°C. |  |
| 3. | 22 day, 27.12.2015, 07:37  (mild metabolic alkalosis= | pH = 7.44 pCO_2_ = 42 mmHg  HCO_3_ = 28.5 mmol/L  pO_2_ = 138 mmHg  BE(ecf) = 4.3 mmol/L | No sedation, basic contact possible to maintain, pupils equal and reactive to light. The patient breathes on her own via the tracheostomy with passive oxygen therapy. Numerous rales above the lungs, considerable amounts of thin mucous secretion sucked off the bronchial tree. Cardiovascularly stable. Temporary tachycardia at a rate of 130 BPM. Betaloc (Metoprololi succinas) administered. Muscle strength weakening still noticeable. |  |
| 3. | 22 day, 27.12.2015, 15:35  (metabolic alkalosis | pH = 7.46 pCO_2_ = 39 mmHg  HCO_3_ = 27.7 mmol/L  pO_2_ = 150 mmHg  BE(ecf) = 3.9 mmol/L |  |  |
| 4. | 23 day, 28.12.2015, 07:36  (respiratory acidosis)  hypodynamic respiratory insufficiency | pH = 7.39 pCO_2_ = 52 mmHg  HCO_3_ = 31.5 mmol/L  pO_2_ = 147 mmHg  BE(ecf) = 6.5 mmol/L | No sedation, basic contact possible to maintain, pupils equal and reactive to light. Muscle strength weakening. The patient breathes on her own via the tracheostomy with passive oxygen therapy. Numerous rales above the lungs, considerable amounts of thin mucous secretion sucked off the bronchial tree. Aflegan (Ambroxoli hydrochloridum) stopped. Cardiovascularly stable. Temporary tachycardia at a rate of 130 BPM. |  |
| 9. | 25 day, 30.12.2015, 07:54  Respiratory acidosis - hypodynamic respiratory insufficiency | pH = 7.33 pCO_2_ = 79 mmHg  HCO_3_ = 41.7 mmol/L  pO_2_ = 179 mmHg  BE(ecf) = 15.8 mmol/L | No sedation, the patient sleepy, breathes via the tracheostomy, cardiovascularly stable. Considerable amounts of mucous secretion sucked off the bronchial tree. | 29.12.2015  CRP: 4.3 mg/l procalcitonin < 0.1 ng/ml Reduction of diuresis. |
| 9. | 26 day, 31.12.2015, 07:56  Respiratory acidosis - hypodynamic respiratory insufficiency  (slightly too low CO_2_ = 5 mmHg) | pH = 7.29 pCO_2_ = 92  HCO_3_ = 44.2 mmHg  pO_2_ = 131 mmHg  BE(ecf) = 17.6 mmol/L | Patient still sleepy, in the morning **respiration again supported in the bilevel positive airway pressure (BPAP) mode**, considerable amounts of mucous secretion sucked off the bronchial tree. Continuous tendency for hypotension. | *Reduction of diuresis: Furosemid (furosemidum).*  *CI: 2.9–2.7* |
| 10. | 26 day, 31.12.2015, 19:14  Mixed respiratory and metabolic alkalosis  – the use of mechanical ventilation | pH = 7.70 pCO_2_ = 30 mmHg  HCO_3_ = 37.7 mmol/L  pO_2_ = 91 mmHg  BE(ecf) = 17.2 mmol/L  **lactates = 1.3 mmol/L** |  |  |
| 11. | 27 day, 01.01.2016, 07:45  (mixed respiratory/metabolic alkalosis)  Normalization of blood gases - the use of mechanical ventilation | pH = 7.66 pCO_2_ = 25 mmHg HCO3=28.2 mmol/L  pO_2_ = 160 mmHg  BE(ecf) = 7.6 mmol/L | Patient conscious, temporarily sleepy, establishes basic contact easily. Mechanical respiration in the bilevel positive airway pressure (BPAP) mode maintained, mucous secretion sucked off the bronchial tree. Patient cardiovascularly stable. | *Reduction of diuresis: Furosemid (furosemidum).* |
| 3. | 27 day, 01.01.2016, 13:52  (as above) | pH = 7.44 pCO_2_ = 39 mmHg  HCO_3_ = 26.5 mmol/L  pO_2_ = 139 mmHg  BE(ecf) = 2.3 mmol/L |  |  |
| 2. | 27 day, 01.01.2016, 21:23 (as above) | pH = 7.47 pCO_2_ = 36 mmHg  HCO_3_ = 26.2 mmol/L pO_2_ = 163 mmHg  BE(ecf) = 2.5 mmol/L |  |  |
| 2. | 28 day, 02.01.2016, 4:16  (metabolic alkalosis) | pH = 7.49  pCO_2_ = 36 mmHg  HCO_3_ = 27.4 mmol/L  pO_2_ = 148 mmHg  BE(ecf) = 4.1 mmol/L | Patient conscious, remains in basic contact. Mechanical ventilation in the bilevel positive airway pressure (BPAP) mode continued, PEEP = 10/3, FiO_2_ = 0.3. Individual rales hearable from the areas above the lungs. Considerable amounts of mucous secretion still sucked off the air passages. | *Reduction of diuresis: Furosemid (furosemidum).* |
| 2. | 29 day, 03.01.2016, 07:55  (metabolic alkalosis) | pH = 7.47 pCO_2_ = 42 mmHg  HCO_3_ = 30.6 mmol/L  pO_2_ = 140 mmHg  BE(ecf) = 6.9 mmol/L | Patient conscious, attempts to answer questions moving her head, still muscle strength very weak. Weakly squeezes both hands on request and moves both feet. |  |
| 12. | 29 day, 03.01.2016, 21:41 | pH = 7.52 pCO_2_ = 36 mmHg  HCO_3_ = 29.4 mmol/L  pO_2_ = 139 mmHg  BE(ecf) = 6.5 mmol/L |  |  |
| 12. | 30 day, 04.01.2016, 07:22 (metabolic alkalosis)  *Interpretation: ↑pCO_2_ caused by decrease in ventilator support;*  *delay in urinary HCO_3_ excretion* | pH = 7.51 pCO_2_ = 37 mmHg HCO_3_ = 29.5 mmol/L  pO_2_ = 151 mmHg  BE(ecf) = 6.5 mmol/L | Patient conscious, remains in basic contact. Muscle strength still very weak. Spontaneous respiration of very low volume, not ensuring elimination of carbon dioxide. CPAP with PS maintained. |  |
| 3. | 30 day, 04.01.2016, 15:08  *(as above)* | pH = 7.44 pCO_2_ = 45 mmHg  HCO_3_ = 30.6 mmol/L  pO_2_ = 126 mmHg  BE(ecf) = 6.4 mmol/L |  |  |
| 3. | 31 day, 05.01.2016, 14:30  (metabolic alkalosis)  *Interpretation:* ↑pCO_2_ caused by ↓ventilator support | pH = 7.44 pCO_2_ = 47 mmHg HCO_3_ = 31.9 mmol/L  pO_2_ = 136 mmHg  BE(ecf) = 7.7 mmol/L | Patient conscious, remains in basic contact. CPAP mode ventilation with PS, FiO_2_ = 0.3. |  |
| 3. | 32 day, 06.01.2016, 14:10 (metabolic alkalosis)  *Interpretation: delay in urinary HCO_3_ excretion* (disproportionally low pCO_2_ of 1–2 mmHg) | pH = 7.46 pCO_2_ = 41 mmHg  HCO_3_ = 29.2 mmol/L  pO_2_ = 158 mmHg  BE(ecf) = 5.4 mmol/L | Patient conscious, it seems that right hand mobility has improved greatly. Also head movements cover wider range. Cardiovascularly stable, fever absent, mechanical ventilation in the CPAP mode with PS, 30% oxygen. Short apnoea temporarily during sleep. |  |
| 2. | 33 day, 07.01.2016, 07:42  (metabolic alkalosis) | pH = 7.47 pCO_2_ = 42 mmHg  HCO_3_ = 30.6 mmol/L  pO_2_ = 157 mmHg  BE(ecf) = 6.9 mmol/L |  |  |
| 5. | 34 day, 08.01.2016, 21:31  Respiratory acidosis caused by reducing ventilator support (slightly too low CO_2_ = 1–2 mmHg) | pH = 7.40 pCO_2_ = 55 mmHg  HCO_3_ = 34.1 mmol/L  pO_2_ = 141 mmHg  BE(ecf) = 9.3 mmol/L | Patient conscious, remains in basic contact, muscle strength still very weak. Assisted PAV respiration, FiO_2_ = 0.3. Patient cardiovascularly stable. |  |
| 5. | 35 day, 09.01.2016, 07:27  (slightly too low CO_2_ = 1–2 mmHg)  Respiratory acidosis caused by reducing ventilator support | pH = 7.39 pCO_2_ = 60 mmHg  HCO_3_ = 36.3 mmol/L  pO_2_ = 152 mmHg  BE(ecf) = 11.3 mmol/L | Patient’s general and neurological condition with no greater alterations. Patient conscious, remains in basic contact. Assisted PAV respiration, FiO_2_ = 0.3. |  |
| 5. | 36 day, 10.01.2016, 07:52  Respiratory acidosis caused by reducing ventilator support  (slightly too low CO_2_ = 2–3 mmHg) | pH = 7.38 pCO_2_ = 67 mmHg  HCO_3_ = 39.6 mmol/L  pO_2_ = 166 mmHg  BE(ecf) = 14.5 mmol/L | Patient in general critical but stable condition. Patient conscious, reacts to commands, sleeps for most of the on-call time. Muscle strength considerable weakened. **No spontaneous activity. Assisted PS respiration, with temporal apnoea periods. Change to SIMV. FiO_2_ = 0.35. CO_2_ = 40–50 mmHg**. |  |
| 1. | 37 day, 11.01.2016, 03:36  Metabolic alkalosis caused by increasedventilatory support | pH = 7.47 pCO_2_ = 45 mmHg  HCO_3_ = 32.8 mmol/L  pO_2_ = 141 mmHg  BE(ecf) = 9.1 mmol/L |  |  |
| 12. | 37 day, 11.01.2016, 22:24  Metabolic alkalosis caused by increased ventilatory support | pH = 7.51mmHg pCO_2_ = 35 mmol/L  HCO_3_ = 27.9 pO_2_ = 120 mmHg  BE(ecf) = 4.9 mmol/L | Patient’s general and neurological condition with no greater alterations. Patient conscious, remains in basic contact. Assisted CPAP respiration, FiO_2_ = 0.35. Patient cardiovascularly stable. | *Reduction of diuresis.* |
| 3. | 38 day, 12.01.2016, 07:59 ormalization of blood gases - the use of appropriate mechanical ventilation | pH = 7.46 pCO_2_ = 41 mmHg  HCO_3_ = 29.2 mmol/L  pO_2_ = 188 mmHg  BE(ecf) = 5.4 mmol/L |  |  |
| 4. | 38 day, 12.01.2016, 20:46  Respiratory acidosis caused by reducing ventilator support | pH = 7.40 pCO_2_ = 51 mmHg  HCO_3_ = 31.6 mmol/L  pO_2_ = 145 mmHg  BE(ecf) = 6.8 mmol/L | Patient conscious, reacts to commands, sleeps for most of the on-call time. Muscle strength considerable weakened. **No spontaneous activity. Assisted PS respiration, FiO_2_ = 0.35. CO_2_ = 40–50 mmHg. Before noon change to TC. CO_2_ levels stable**. |  |
| 5. | 39 day, 13.01.2016, 13:52  Respiratory acidosis caused by reducing ventilator support  (slightly too low CO_2_ = 2–3 mmHg) | pH = 7.40 pCO_2_ = 55 mmHg  HCO_3_ = 34.1 mmol/L  pO_2_ = 136 mmHg  BE(ecf) = 9.3 mmol/L |  |  |
| 5. | 39 day, 13.01.2016, 21:13  Respiratory acidosis - rise the carbon dioxide caused by spontaneously breathing patient | pH = 7.39 pCO_2_ = 61 mmHg  HCO_3_ = 36.9 mmol/L  pO_2_ = 170 mmHg  BE(ecf) = 11.9 mmol/L | Patient conscious, remains in basic contact. Assisted TC respiration, FiO_2_ = 0.3, **later the patient breathes on her own, with passive oxygen therapy**. Patient cardiovascularly stable. | *Chronic respiratory acidosis.* |
| 13. | 40 day, 14.01.2016, 21:26  Mixed respiratory acidosis and metabolic alkalosis | pH = 7.45 pCO_2_ = 52 mmHg  HCO_3_ = 36.9 mmol/L  pO_2_ = 71 mmHg  BE(ecf) = 12.1 mmol/L | Patient conscious, reacts to commands, sleeps for most of the on-call time. Muscle strength considerable weakened. No spontaneous activity. The patient has been breathing on her own for a day, with no visible respiratory effort. CO_2_ = 55–65 mmHg. | *Chronic respiratory acidosis.* |
| 13. | 41 day, 15.01.2016, 14:48  (metabolic alkalosis)  (slightly too low CO_2_ = 1–2 mmHg) | pH = 7.48 pCO_2_ = 51 mmHg  HCO_3_ = 38.0 mmol/L  pO_2_ = 97 mmHg  BE(ecf) = 14.5 mmol/L | Patient conscious, sleepy. Spontaneous respiration. |  |
| 13. | 42 day, 16.01.2016, 14:37  (metabolic alkalosis) | pH = 7.47 pCO_2_ = 53 mmHg  HCO_3_ = 38.6 mmol/L  pO_2_ = 79 mmHg  BE(ecf) = 14.9 mmol/L | Spontaneous respiration via the tracheostomy with no with passive oxygen therapy. |  |
| 13. | 43 day, 17.01.2016, 15:57  (metabolic alkalosis) | pH = 7.46 pCO_2_ = 54 mmHg  HCO_3_ = 38.4 mmol/L  pO_2_ = 79 mmHg  BE(ecf) = 14.6 mmol/L | Spontaneous respiration via the tracheostomy with no with passive oxygen therapy. Cardiovascularly stable. |  |
| 13. | 44 day, 18.01.2016, 13:39  (mixed respiratory acidosis/metabolic alkalosis) | pH = 7.46 pCO_2_ = 53 mmHg  HCO_3_ = 37.7 mmol/L  pO_2_ = 75 mmHg  BE(ecf) = 12.2 mmol/L |  |  |
| 14. | 45 day, 19.01.2016, 08:06  (respiratory acidosis) | pH = 7.42 pCO_2_ = 54 mmHg  HCO_3_ = 35.0 mmol/L  pO_2_ = 99 mmHg  BE(ecf) = 10.5 mmol/L | Patient conscious, reacts to commands. Has been breathing on her own for the following day, with no visible respiratory effort, parameters of respiratory gas exchange within normal range. Patient cardiovascularly stable. | *Chronic respiratory acidosis.* |
| 1. | 46 day, 20.01.2016, 07:45  (metabolic alkalosis, slow blood gases normalization)s | pH = 7.44 pCO_2_ = 48 mmHg  HCO_3_ = 32.6 mmol/L  pO_2_ = 90 mmHg  BE(ecf) = 8.4 mmol/L | At present patient conscious, contact with patient difficult. Spontaneous respiration via the tracheostomy tube, patient cardiovascularly stable, gastroenteric tube feeding, industrial diet. Fever absent. Patient transferred to the Clinic of Neurology. |  |
