# Supplementary material for: Leigh syndrome in individuals bearing m.9185T>C MTATP6 variant. Is hyperventilation a factor which starts its development?
Source: Metab Brain Dis. 2017 Nov 7;33(1):191–9. doi: 10.1007/s11011-017-0122-1 (PMC5769826; doi:10.1007/s11011-017-0122-1)
Supplement: Supplementary file 2 — (DOCX 115 kb) [file 11011_2017_122_MOESM2_ESM.docx]

Supplementary Table2. Characteristics of 81 individuals with m.9185T>C variant in *MTATP6*, this study and literature data.

| Family | Patient, sex | Age of onset/exam | Heteroplasmy level | Diagnosis | Disease course; last known age | Reference |
| --- | --- | --- | --- | --- | --- | --- |
| 1. | (p) F | 5 | >97% (B, M, U) | LS | improvement, 9 y | [1] this study |
|  | (m) F | 34 | >97% (B) | LS | improvement, 35 y |  |
|  | (gm) F | 61 | 45% (B), 55% (U) | carrier | - |  |
|  | (s) F | 32 | 15% (B, U) | carrier | - |  |
| 2. | (p) M | 9 | >97% (B) | LS | fulminant; D (9 y) | [2] Pronicka et al. 2016* |
|  | (m) F | 40 | >97% (B) | carrier? Alzheimer (*PSEN1*) | (severe), 43 y |  |
|  | (s) M | 17 | >97% (B) | carrier | - |  |
| 3 | (p) M | 0.5 | nd | LS | D (nd) | [3] Danqun et al 2015 (abstract only) |
| 4 | (p) M | childhood | 100% | MND | benign, nd | [4] Brum et al. 2014 |
|  | (m) F | nd | nd | congenital deafness, stroke | D (48 y) |  |
|  | (s3) M | childhood | 100% | polyneuropathy, epi | benign, nd |  |
|  | (s1) M | 34 | nd | LS | D (34 y) |  |
|  | (s2) F | 16 | nd | LS | D (16 y) |  |
| 5  family 1 | Pt 1 (F); IV-5 | 18 | 98 (B), 78 (U), 100 (Bc), 100 (H), 99 (F), 100 (M); | periodic paralysis, gait disorder | slowly progressive*; 43 y | [5] Auré et al. 2013 |
|  | Pt 2 (F); V-5 | 13 | 89 (B), 99 (U), 100 (Bc), 100 (H), 99 (F) | periodic paralysis, polyneuropathy | slowly progressive*; 24 y |  |
|  | Pt 3 (F); V-12 | 12 | 99 (B), 88 (Bc), 96 (H), 98 (M) | periodic paralysis, polyneuropathy | slowly progressive*; 25 y |  |
|  | III-6 (F) | 60 | 83 (B), 100 (U), 100 (Bc), 35 (H), 100 (F), 100 (M) | severe gait imbalance | 79 y |  |
|  | IV-2 (F) | nd | 100 (B), 74 (U), 95 (Bc), 77 (H) | periodic paralysis | nd |  |
|  | IV-3 (F) | nd | 53 (B), 94 (U), 85 (Bc), 81 (H) | periodic paralysis | nd |  |
|  | IV-4 (M) | nd | 100 (B), 98 (U), 99 (Bc), 98 (H) | periodic paralysis | nd |  |
|  | IV-6 (F) | nd | 87 (B), 74 (U), 67 (Bc), 67 (H) | Carrier | Asymptomatic |  |
|  | IV-16 (M) | nd | 99 (B), 89 (U), 99 (Bc), 97 (H) | periodic paralysis | nd |  |
|  | V-3 (M) | nd | 100 (B), 100 (U), 100 (Bc), 78 (H) | periodic paralysis | nd |  |
|  | V-9 (F) | nd | 82 (B), 94 (U), 98 (Bc), 92 (H), 100 (M) | (MELAS due to m.3271T>C in *MTTL1*) | (D, 15 y); |  |
|  | V-13 | nd | 99 (Bc), 99 (H) | periodic paralysis | nd |  |
|  | V-18 | nd | 73% (B), 71 (U) | pes cavus (?) | asymptomatic (?) |  |
| 6  family 2 | Pt 4 (F) | 11 | 100% | periodic paralysis, polyneuropathy | slowly progressive; 35 y |  |
|  | Pt 5 (M) | 11 | 100% | periodic paralysis, polyneuropathy | slowly progressive; 20 y |  |
| 7  family 3 | Pt 6 (M) | 27 | 100% | periodic paralysis, polyneuropathy | slowly progressive; 30 y |  |
| 8 | (s1) M | adolescence | 98% (M) | SCA | 58 y | [6] Pfeiffer i wsp. 2012  (Pyromark software for heteroplasmy) |
|  | (s2) F | 34 | 99% (B) | SCA | 55 y |  |
|  | (s3) M | 32 | 99% (B) | SCA | 51 y |  |
|  | (s4) F | nd | nd | SCA | 49 y |  |
|  | (s5) F | 37 | 95% (B) | SCA | 43 y |  |
| 9 (A) | (gm) I-1 F | 80 | 88% (B) | nd |  | [7] Pitceathly et al 2012 |
|  | (ms) II-1 F | 57 | 100% (B) | CMT t.2 |  |  |
|  | (ms) II-5 M | 47 | 100% (B) | CMT t.2 |  |  |
|  | (s) III-5 | 16 | 100% (B, M) | LS (suspicion) | rapid decline, D (18 y) |  |
|  | (s) III-6 M | 27 | 100% (B, M) | CMT t.2 |  |  |
|  | (p) III-8 M | 21 | 100% (B, U) | CMT t.2 |  |  |
|  | (r) IV-2 | 11 | 100% (M) | Nd |  |  |
|  | (r) IV-1 | 9 | nd | LS (suspicion) | sudden decline, D (9 y) |  |
| 10 (B) | II-8 M | 60 | 100% (B) | CMT t.2 |  |  |
|  | III-3 F | 49 | 80% (B) | MND |  |  |
|  | III-5 F | 48 | 73% (B) | CMT t.2 |  |  |
|  | III-6 F | 46 | 100% (B) | CMT t.2 |  |  |
|  | IV-3 F | 19 | 100 (B) | CMT t.2 |  |  |
|  | IV-4 F | 21 | 39% (B) | carrier |  |  |
| 11 (C) | III-5 F | 48 | 75% (B) | nd |  |  |
|  | III-13 M | 45 | 100% (B) | CMT t.2 |  |  |
|  | IV-1 M | 18 | 45% (B) | carrier |  |  |
|  | IV-2 M | 21 | 100% (B) | CMT t.2 |  |  |
| 12. (D) | III-1 M | 45 | 92% (B) | CMT t.2 |  |  |
| 13 |  | 3 | 100% | LS | subacute onset, recovery, recurrent LS; 8 y | [8] Saneto i wsp. 2010 |
|  | (m) | adult | 30% | carrier | - |  |
| 14 | (p) M | 8.5 | >90% (B, F, LB) | LS | subacute-onset, D | [9] Castagna et al 2007 |
|  | (s1) M | 7 | >90% (B, F, LB) | LS | sudden onset, recovery, nd |  |
|  | (s2) M |  | 17% (LB) | carrier | - |  |
|  | (m) | adolescence | 86% | CMT | ataxia, weakness, nd |  |
|  | (ms1) F | 3 | 85% | peripheral neuropathy | slowly progressive, 26 y |  |
|  | (mr) F | 7 | 90% (all tissues) | LS (at autopsy) | fluctuating, hyperventilation episodes, D (10 y) |  |
|  | (mr) M | 10 | 91% (all tissues) | intermitent ataxia | improvement, 11 y |  |
|  | (gm) F | adult | 52% (LB) | carrier | - |  |
|  | (ms2) F | adult | 81% (B) | carrier | - |  |
| 15 | II-3 F | 13 | 100% | NARP | 46 y | [10] Childs et al. 2007 |
|  | II-4 F | 5 | 100% | NARP | 45 y |  |
|  | III-1 M | 21 | 100% | peripheral neuropathy (pes cavus, absent ankle reflexes) | mildly affected |  |
|  | III-2 M | 19 (6) | 100% | LS, normal lactate level  (MRI normal at 13 y) | acute onset, partial improvement, panic attacks; 24 y |  |
|  | III-3 M | 4 | 100% | NARP | 20 y |  |
|  | III-4 F | childhood | 100% | peripheral neuropathy | mildly affected |  |
|  | III-5 M | childhood | 100% | peripheral neuropathy | mildly affected |  |
|  | III-6 M | 7 (1) | 100% | LS, HCM, IPPV | acute onset, D after 3 weeks (7 y) |  |
|  | III-7 F | childhood | 100% | peripheral neuropathy | mildly affected |  |
|  | III-8 M | 11 (3) | 100% | LS, increased lactate level, IPPV for 3 weeks | subacute onset, partial improvement; 22 y |  |
|  | III-9 M | childhood | 100% | peripheral neuropathy | mildly affected |  |
|  | III-10 M | childhood | 100% | peripheral neuropathy | mildly affected |  |
|  | III-11 M | 12 (1) | 100% | LS | acute onset, good outcome but decreased IQ; 18 y |  |
|  | III-12 F | 17 (8) | 100% | LS, increased lactate level, IPPV for 4 weeks | subacute onset, partial improvement; 24 y |  |
|  | III-13 M | 1 | 100% | NARP | 21 y |  |
| 16 | (p) M | 7 | >97% (B, M) | LS | nd | [11] Moslemi et al 2005 |
|  | (m) F | adult | 85% | carrier | - |  |

LS, Leigh syndrome; CMT t.2, Charcot-Marie-Tooth syndrome type 2; SCA, spinocerebellar ataxia; MND, motor neuron disease; MELAS, mitochondrial encephalopathy, lactic acidosis, stroke-like episodes ; NARP, neurogenic atrophy, retinitis pigmentosa; p, proband; m, mother, gm, grandmother; s, sibling, r, relative; F, female; M, male; B, blood, leukocytes; M, muscle; U, urine; Bc , buccal cells; LB, lymphoblasts; F, fibroblasts; nd, not determined or not known.

* precipitating factors: effort, prolonged sitting, anxiety, cold or hot weather, rest after effort
