# Supplementary material for: Leigh syndrome in individuals bearing m.9185T>C MTATP6 variant. Is hyperventilation a factor which starts its development?
Source: Metab Brain Dis. 2017 Nov 7;33(1):191–9. doi: 10.1007/s11011-017-0122-1 (PMC5769826; doi:10.1007/s11011-017-0122-1)
Supplement: Supplementary file 3 — (DOCX 113 kb) [file 11011_2017_122_MOESM3_ESM.docx]

Supplementary Table 3. Characteristics of LS development in 21 individuals with m.9185T>C *MTATP6* variant, this study and literature data.

| Patient (sex); references | Initial symptoms | LS onset | Outcome |
| --- | --- | --- | --- |
| MasW (F) | Sslightly slurred slow speach during early childhood | 5 y: during infection, coughing, tachypnoe, sighs, ptosis, unsteady gait. | 9 y: without symptoms, MRI improvement |
| MasI (F) | fatigability in childhood | 34 y: deterioration during infection, hyperventilation | partial recovery |
| PęW (M); [Pronicka et al] | sluggish speech in childhood, tachypnea episodes | 9 y: after 3 day febrile illness: hyperventilation, unstady gait, dysarthria | death within 1 month |
| III-2 (F) [Child et al ] | 6 y: abnormal gait, pyramidal signs,;  12 y: MRI normal | 19 y: acute onset, diplopia, dysathria, loss of consciousness | partial recovery, depression, panic attacks |
| III-6 (F) [Child et al] | 1 y: vomiting episodes, developmental delay pyramidal signs, peripheral neuropathy | 7 y: acute exacerbation, ptosis, unconsciousness, bulbal signs | death within 3 weeks |
| III-8 (F) [Child et al] | 3 y: developmental delay, failure to thrive; seizures, ataxia, pyramidal signs | 11 y: subacute onset with ataxia, ptosis, bulbal weakness | 22 y: recovery; cognitive decline |
| [Child et al] | 1 y: failure to thrive, pyramidal signs, peripheral neuropathy; ataxia (III-11) | 12 y: acute onset, weakness, ophthalmoplegia, respiratory failure | 18 y: cognitive decline, ataxia |
| [Child et al] | 8 y: foot deformity, mild learning difficulties (III-12) | 17 y: ophthalmoplegia, bulbal weakness, seizures | Partial recovery |
| [3] Danqun et al 2015 (abstract ) | No | Two episodes (4 and 7 mo); abnormal respiration, pulmonary oedema, | death, ND |
| [3] Danqun et al 2015 (abstract ) | psychomotor retardation, hypotonia | 8 mo, abnormal respiration, pulmonary oedema, mechanical ventilation | death , 8 mo |
| [4] Brum et al. 2014 | ND | ND | Death by suicide at 16 y |
| [4] Brum et al. 2014 | ND | Respiratory failure at age 34 y | Death |
| [7] III-5 Pitceathly 2012 | ND | 16 y, rapid decline after viral illness, cortical blindness | Death |
| [7] IV-1 Pitceathly 2012 | 6 y, hyperreflexia, learning difficulties, mobility unaided, last seen at 8 y | 9 y, rapid deterioration after febrile viral-like illness | Death |
| [8] Saneto i wsp. 2010 | No | 3 y, 8 10 y:exacerbations corresponded to febrile illness or summer heat | Partial recovery |
|  | No (III-7, proband) | 8.5 y, deterioration during a febrile illness | Partial recovery |
| [9] Castagna et al 2007 | ND (III-8, proband's older brother) | 7 y, ataxia following a febrile viral illness | Death within 2 weeks |
| [9] Castagna et al 2007 | Seizures in infancy; borderline IQ testing. (II-2) | 7y, ‘‘encephalitis’’; 10 y, weakness, attacks of hyperventilation, respiratory failure; LS on autopsy | Death after 4 months |
| [9] Castagna et al 2007 | ND (II-5, Figure) | ND | Death |
| [9] Castagna et al 2007 | No (II-5) | 7 y, lassitude, partial ptosis , ataxia for 4 mo following a flu-like illness | Death |
| [11] Moslemi et al 2005 | No | 7 y, suddenly: ptosis, ophthalmoparesis, muscular hypotonia | Clinical normalization , MRI improvement |
